# Ground-Based Optical Measurements at European Flux Sites: A Review of Methods, Instruments and Current Controversies

**DOI:** 10.3390/s11087954

**Published:** 2011-08-12

**Authors:** Manuela Balzarolo, Karen Anderson, Caroline Nichol, Micol Rossini, Loris Vescovo, Nicola Arriga, Georg Wohlfahrt, Jean-Christophe Calvet, Arnaud Carrara, Sofia Cerasoli, Sergio Cogliati, Fabrice Daumard, Lars Eklundh, Jan A. Elbers, Fatih Evrendilek, Rebecca N. Handcock, Joerg Kaduk, Katja Klumpp, Bernard Longdoz, Giorgio Matteucci, Michele Meroni, Lenoardo Montagnani, Jean-Marc Ourcival, Enrique P. Sánchez-Cañete, Jean-Yves Pontailler, Radoslaw Juszczak, Bob Scholes, M. Pilar Martín

**Affiliations:** 1 DIBAF—University of Tuscia, Via S.C. de Lellis, 01100 Viterbo, Italy; E-Mail: arriga@unitus.it; 2 Department of Geography, University of Exeter, Rennes Drive, Exeter, EX4 4RJ, UK; E-Mail: Karen.Anderson@exeter.ac.uk; 3 School of GeoSciences, University of Edinburgh, West Mains Road, Edinburgh EH9 3JN Scotland, UK; E-Mail: Caroline.Nichol@ed.ac.uk; 4 DISAT—University of Milan-Bicocca, piazza della Scienza 1, 20126 Milano, Italy; E-Mails: micol.rossini@unimib.it (M.R.); sergio.cogliati@unimib.it (S.C.); michele.meroni@jrc.ec.europa.eu (M.M.); 5 CRI—Fondazione E. Mach, Viote del Monte Bondone, Trento, Italy; E-Mail: loris.vescovo@iasma.it; 6 Institut für Ökologie, Universität Innsbruck, Sternwartestr. 15, 6020 Innsbruck, Austria; E-Mail: georg.wohlfahrt@uibk.ac.at; 7 CNRM/GAME–Meteo-France, CNRS, URA 1357, 42 avenue Coriolis, 31057 Toulouse Cedex 1, France; E-Mail: jean-christophe.calvet@meteo.fr; 8 Fundacion CEAM, Parque Tecnologico, Calle C.R. Darwin, Paterna, SP-46980, Valencia, Spain; E-Mail: arnaud@ceam.es; 9 Forest Research Centre, School of Agriculture, Technical University of Lisbon, Tapada da Ajuda, 1349-017, Lisboa, Portugal; E-Mail: sofiac@isa.utl.pt; 10 Laboratoire de Météorologie Dynamique, Ecole Polytech., Palaiseau, France; E-Mail: fabrice.daumard@lmd.polytechnique.fr; 11 Department of Earth and Ecosystem Sciences, Lund University, Sölvegatan 12, SE-223 62, Lund, Sweden; E-Mail: lars.eklundh@nateko.lu.se; 12 Alterra, ESS-CC Group, PO Box 47, 6700 AA Wageningen, The Netherlands; E-Mail: Jan.Elbers@wur.nl; 13 Department of Environmental Engineering, Abant Izzet Baysal University, Gölköy Campus, 14280 Bolu, Turkey; E-Mail: fevrendilek@yahoo.com; 14 CSIRO, Private Bag 5, Wembley, WA, 6913, Australia; E-Mail: rebecca.handcock@csiro.au; 15 Department of Geography, University of Leicester, Leicester LE1 7RH, UK; E-Mail: jk61@le.ac.uk; 16 INRA, Grassland Ecosystem Research Unite, UR874, 63100 Clermont Ferrand, France; E-Mail: katja.klumpp@clermont.inra.fr; 17 INRA, Ecologie et Ecophysiologie Forestière, UMR1137, Centre de Nancy, F-54280 Champenoux, France; E-Mail: longdoz@nancy.inra.fr; 18 CNR, Institute for Agricultural and Forestry Systems in the Mediterranean, Via Cavour, 4-6 I-87036, Rende, Italy; E-Mail: giorgio.matteucci@isafom.cs.cnr.it; 19 European Commission, DG-JRC, Institute for Environment and Sustainability, Monitoring Agricultural Resources Unit, Via Fermi 2749, 21027, Ispra, Varese, Italy; E-Mail: michele.meroni@jrc.ec.europa.eu; 20 Forest Services and Agency for the Environment, Autonomous Province of Bolzano, Bolzano, Italy; E-Mail: leonar@inwind.it; 21 Faculty of Science and Technology, Free University of Bolzano, Bolzano, Italy; E-Mail: leonar@inwind.it; 22 DREAM-CEFE-CNRS, UMR5175, BP5051, 1919 Route de Mende, F-34293, Montpellier Cedex 5, France; E-Mail: jean-marc.ourcival@cefe.cnrs.fr; 23 IAA–CSIC, c/Camino Bajo de Huétor, 50, Granada 18008, Spain; E-Mail: enripsc@ugr.es; 24 Laboratoire d’Ecologie Végétale, CNRS URA 1492 Bât. 362, Université Paris Sud-Orsay, 91405 Orsay Cedex, France; E-Mail: jean-yves.pontailler@u-psud.fr; 25 Dept. of Meteorology, Poznan University of Life Sciences, Piatkowska 94, 60-649 Poznan, Poland; E-Mail: radjusz@up.poznan.pl; 26 CSIR, Ecosystem Processes and Dynamics, PO Box 395, 0001 Pretoria, South Africa; E-Mail: bscholes@csir.co.za; 27 CCHS—CSIC, Albasanz 26-28, Madrid 28037, Spain; E-Mail: mpilar.martin@cchs.csic.es

**Keywords:** optical measurements, sensors, protocol standardisation, European flux networks

## Abstract

This paper reviews the currently available optical sensors, their limitations and opportunities for deployment at Eddy Covariance (EC) sites in Europe. This review is based on the results obtained from an online survey designed and disseminated by the Co-cooperation in Science and Technology (COST) Action ESO903—“*Spectral Sampling Tools for Vegetation Biophysical Parameters and Flux Measurements in Europe*” that provided a complete view on spectral sampling activities carried out within the different research teams in European countries. The results have highlighted that a wide variety of optical sensors are in use at flux sites across Europe, and responses further demonstrated that users were not always fully aware of the key issues underpinning repeatability and the reproducibility of their spectral measurements. The key findings of this survey point towards the need for greater awareness of the need for standardisation and development of a common protocol of optical sampling at the European EC sites.

## 1. Introduction: The Need for a Standardized Spectral Measuring System for Deployment at Eddy Covariance Sites

Understanding the impacts of climate change on ecosystem structure and functions and predicting ecosystem responses to climate change is one of science’s great challenges. In a changing climate, it is critical to understand the dynamics of ecosystem carbon fluxes through monitoring of vegetation seasonal changes that depend on the complex flux responses to environmental drivers, mainly linked to soil water, temperature, light and nutrient availability. Present approaches to understanding ecosystem carbon exchange have been made possible through the direct measurements of carbon dioxide (CO_2_) and water (H_2_O) fluxes at eddy covariance (EC) sites and through indirect modelling approaches for predicting photosynthetic function. However the restricted spatial representativeness of EC fluxes and the lack of several inputs at required spatial and temporal scales for running the models limit the ecological studies based on these approaches. Remote sensing (RS) offers a unique opportunity to address this issue by providing a method for monitoring ecosystems at synoptic temporal and spatial scales through measurements of carbon-related spectral response: from local scale *in situ* measurements to the global scale by integrating RS data (e.g., MODIS) into ecological models [1–6].

The use of optical RS instruments directly mounted on EC towers can be considered as an important step towards addressing the scaling issue because it plays a crucial role for spatial extrapolation of *in situ* biophysical parameters of vegetation (e.g., phytomass, biomass, LAI, chlorophyll and nitrogen content). In this sense it facilitates process-based modelling, validation, and prediction of CO_2_ and H_2_O fluxes at regional and global scales [7–9] and allows plant photosynthesis to be quantified and monitored temporally [10]. In the last decade several efforts have been initiated by international research groups with the goal of fusing EC data and tower-based optical measurements [11]. Existing hyperspectral systems have been installed at EC sites following different protocols: using a dual beam automatic system fixed on the tower [12], on mobile platforms [13], adopting rotating hemispherical spectrometers [14] or automated systems for multiangular observations of canopy reflectance [15–17]. Spectral properties of vegetation can be also measured by mounting multispectral sensors on flux towers and include instruments such as the Cropscan (Cropscan, USA) or CIMEL (Cimel Electronique, FR, Europe) radiometers. These systems incorporate discrete distinct wavebands, each sensitive to a specific vegetation parameter. Today routine measurements of vegetation indices such as the NDVI (Normalized Difference Vegetation Index), PRI (Photochemical Reflectance Index) and WBI (Water Band Index) are made possible using custom built 2- and 4-band radiometers, and currently marketed by Skye Instruments in the UK. Work by Huemmrich and colleagues [18] demonstrated that reflectance in the broad bands of photosynthetically active radiation (PAR; 400–700 nm) and near-infrared (NIR; 700–3,000 nm) may be used to approximate the NDVI with a “broadband NDVI”. To calculate this broadband NDVI four-component net radiometers (e.g., CNR-1/CNR-4, Kipp&Zonen, NL, Europe; LP NET14, Delta-OMH, IT, Europe; NR01/RA01, Hukseflux, NL, Europe) together with PAR sensors can be used. Several groups have adopted this approach and have developed their own sensors centered on these NDVI bands [19,20] while recently Ruy *et al.* [21] showed the possibility to use light emitting diodes (LEDs) for monitoring vegetation reflectance in narrow spectral bands.

The accuracy of these studies is controlled largely by the radiometric and spectral characteristics inherent in the sensors themselves. Spectral range of the sensors, channel position, and their resolution (Full Width at Half Maximum, or FWHM) usually vary from sensor to sensor, and with the sensor set-up (e.g., height above the canopy, mounting angle, nadir or off-nadir view, field of view-FOV) and local conditions (e.g., fraction of diffuse to direct radiation). In addition to this, some of the products derived from these sensors (e.g., NDVI) are significantly affected by the specific placement and spectral bandwidths of the component measurements in red and near infrared regions [22,23].

One of the main scientific challenges is the effective integration of optical sampling at EC sites with other measurements, *i.e.*, the scale-appropriateness of the spectral measurements relative to the spatial sampling of EC. It is therefore necessary to understand the extent of the optical footprint and how a system can be configured to best sample the flux footprint. For cross-comparison of different EC sites with different vegetation types and distinct spectral properties it is fundamental to understand the optimal sensor set-up which can also be deployed at a variety of sites such that the measurements are both reproducible and comparable. In this context currently a strong international effort has been initiated to address these issues within both the flux and spectral communities. Two international groups exist:

1. In the EC domain, FLUXNET is a global network of EC sites where the exchanges of carbon dioxide, water vapor, and energy between the biosphere and atmosphere are continuously measured (http://www.fluxnet.ornl.gov/fluxnet/index.cfm) [24].
2. In the spectral domain, both SpecNet (http://specnet.info) [25] and the European equivalent COST Action ES0903 (EUROSPEC) (http://cost-es0903.fem-environment.eu/) initiatives both focus on the identification of the scientific requirements for optical measurement systems which are reliable, scale-appropriate, cost-effective and validated for deployment on flux towers globally.

There is not yet a common and standardised measurement protocol for the use of optical systems towards the aim of deploying spectral measurement systems at EC sites, although a series of research groups are attempting to investigate the data quality requirements for such studies [26]. As a result, instrument selection for these purposes has been typically performed on an *ad-hoc* basis by individual groups working at only a few flux tower sites. Therefore spectral measurements protocols vary from site-to-site and often lack traceability to national standards and the use of these data for cross-comparison studies or for scaling-up observations is currently very limited.

The overall aim of this paper is to discuss opportunities and limitations of current optical sensors and methods used across the European EC network and to provide some recommendations on how optical sampling should be carried out. Specific objectives of our study were to determine the type of instruments being used (e.g., hyperspectral, broadband, narrowband) and to determine the mode of deployment (e.g., reflectance quantity measured and sensor’s configuration and calibration status). This review was based on the responses obtained from the questionnaire designed during the first year of activities of COST Action ES0903.

## 2. Method: Design of a Questionnaire on Spectral Measurements at European EC Sites

**Figure 1.**
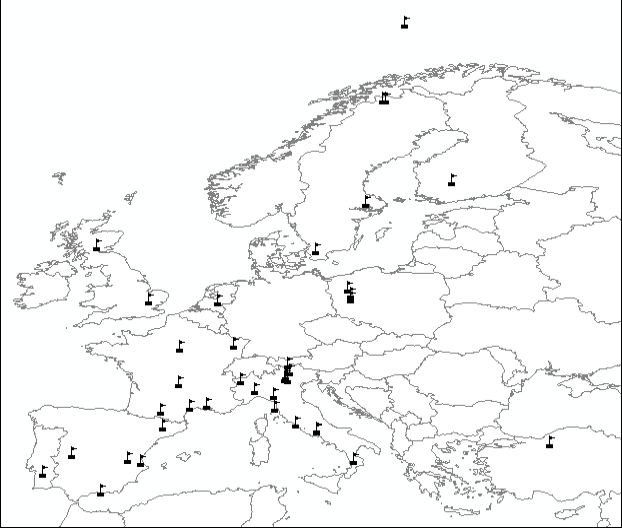
Location of eddy covariance sites of COST countries in Europe working on spectral measurements that filled the questionnaire.

From July 2010 to February 2011 the management committee of the COST Action ES0903 distributed an online survey to scientists operating EC sites within the European community (http://tinyurl.com/EUROPSEC-survey). The questionnaire was designed to establish an optimum scenario for spectral measurements at flux towers by providing answers to the most relevant scientific questions related to optical sampling. In order to do that and to give recommendations on optical sensors to use at the EC towers, it was necessary to know which instruments (hyperspectral, multispectral, broadband, narrowband) and methods of data acquisition (conical, bi-conical; single or dual sensor head) are being used, what quantity is measured (e.g., reflectance, radiance, irradiance), and how sensors and data are calibrated (e.g., traceability to international standards). The structure of the questionnaire was organized into four sections. Section 1 contained questions focused on obtaining general information on the eddy-covariance sites (name, location, ecosystem type, management activity) and information about the research teams working at each site. A suite of questions were included to collect information about the research team’s experience in collecting spectral measurements. The core section of the questionnaire was aimed at gathering information concerning the instruments and measurements, the sensor(s) and set-ups (including the sampled spectral quantities, how often instruments and data were calibrated, and information on spectral and multi-angular configurations of used sensors). The final section referred to the specific applications of the spectral measurements (including estimation of biophysical parameters and up scaling activities using satellite data). The questionnaire was pre-tested by members of the COST Action ES0903 to confirm that the respondents did not have difficulties in understanding all of the questions and also to evaluate the possibility of including new pre-defined answers. This review is based on the responses to the COST questionnaire obtained from groups that are working at 40 flux tower sites in Europe (Figure 1 and Table 1) with two additional groups working in Africa and Australia (both of which are non-COST country members).

## 3. Results

### 3.1. Flux Site Main Characteristics

Questionnaire were received responses from researchers working at 40 different eddy covariance sites located across the COST countries (Figure 1): one site was located in Austria, one in Denmark, one in Finland, six in France, twelve in Italy, three in Poland, one in Portugal, five in Spain, four in Sweden, one in The Netherlands, one in Turkey and two in the United Kingdom. Also two researchers of EC sites located in countries outside Europe (Africa and Australia) that signed an agreement with the COST Action filled in the questionnaire.

The majority of sites (42.5%, 17 sites) were located in forested areas: seven of which were in evergreen needle leaf forests, six in deciduous broadleaf forests, three in evergreen broadleaf forests and one in mixed forests. The remaining sites included eight grasslands, five croplands, six wetlands, two open scrublands, one open savanna, one woody savanna and one savanna. Respondents could select more than one checkbox, so percentages may add up to more than 100%.

Most of the above EC sites (67.5%, 27 sites) were included in the CarboEurope-IP network from 2004 to 2008 and continued their activities either through national funding (e.g., CarboItaly project in Italy) or through the follow-up IMECC project (http://imecc.ipsl.jussieu.fr/). Nine sites reported that they had been monitoring mass and energy fluxes by EC techniques for more than ten years, thirteen sites from 5 to 10 years and eighteen sites for less than 5 years.

### 3.2. Experience in Spectral Measurements

Of the forty sites considered, twelve had collected spectral data for more than 5 years, eight sites from 3 to 5 years, fifteen sites from 1 to 3 years and five sites less than 1 year. Of these, thirty-eight sites are still making both optical and flux measurements, and only two sites have stopped their measurements.

In terms of support for spectral data collection, 65% (26 sites) of the research groups reported that they had access to specialist staff to manage and analyze data from optical sensors. At these sites, the technical and scientific knowledge about spectral measurements was mainly acquired throughout undergraduate and/or postgraduate studies in related subjects, and sometimes through private studies. The majority of groups (60%, 24 sites) declared to have experience in optical sampling at both leaf and canopy level, in PRI and NDVI monitoring and in remote sensing data analysis. Some teams also mentioned their experience in fluorescence measurements. For those sites (16 sites) who declared not to have a remote sensing specialist in their research group, the investigators learned by themselves to use optical instruments (15 sites) or collaborated with other teams having a background in remote sensing (1 site).

The majority of the respondents (82.5%, 33) knew about the activities of SpecNet [25], but only three sites [Hyytiälä (Finland), Monte Bondone and Torgnon (Italy)] were directly involved in the network. However, when we asked about the potential interest of joining SpecNet, only two sites were not interested in joining the network. Regarding data sharing, just over half (56%) of the respondents were interested in sharing their data under specific agreements, while only 14% of respondents were open to share their data to without restrictions. The remaining respondents (30%) did not answer this question.

### 3.3. Radiance, Irradiance and Reflectance Measurements

A wide range of sensors for optical measurements are now commercially available, and these can typically be classified into three categories: (1) broad-band multispectral sensors; (2) narrow-band multispectral sensors; and (3) hyperspectral sensors. The main distinction between these is their spectral bandwidth across the sampled spectrum. The broad-band multispectral sensors capture data in a few wavelength channels, spanning from tens to hundreds of nanometers across the electromagnetic spectrum. Some multispectral instruments can also be built to offer fine spectral resolution data (e.g., bandwidth < 10 nm) in a few targeted bands—in this paper we will refer to these as being “fine spectral resolution multispectral” instruments. Hyperspectral sensors, on the other hand, measure in very narrow and contiguous (overlapping) channels and can be used to provide more detailed information in the wavelength domain.

Through the responses collected by the questionnaire, it was possible to draw up a summary table describing the sensors and set-ups mainly used at the EC sites (Table 1). As reported in Table 1 the majority of EC sites used multispectral sensors (e.g., Kipp&Zonen) or fine spectral resolution multispectral sensors (e.g., Skye, Cropscan, CIMEL) while only five sites reported installation of hyperspectral sensors.

Optical sensors can be also classified as “single-beam” (S) or “dual-beam” (D) instruments. An instrument configuration is known as “single-beam” when the same detector/instrument is used to measure the spectral radiance of a target and that incident upon it. When two sensors are employed to measure the target and the incident irradiance simultaneously the instrument configuration is referred to as “dual-beam”. The review by Milton *et al.* [34] discusses the relative merits of each of these configurations. Configuration mainly used for two and 4-channel radiometers and fine spectral resolution multispectral sensors is dual-beam while for hyperspectral sensors the mode used is user-dependent and is strictly related to the characteristics of the system (Part 3.4.3).

For those researchers working with reflectance factor quantities (24 sites), we posed further questions in order to understand which quantities were being measured, according to Schaepman-Strub’s nomenclature [35]. Only 42% (10 sites) had read the Schaepman-Strub *et al.* paper [35] and were informed about the range of distinct “reflectance” quantities. The Bi-Hemispherical Reflectance factor (BHR) and Hemispherical-Conical Reflectance Factor (HCRF) were most often measured (Table 1). All the respondents considered the use of standardised nomenclature to be important, but many did not comment on whether they correctly applied it. This is an important issue because the correct application of the nomenclature when publishing work is critical to the reproducibility of the results. Without recognition of the central importance of nomenclature in the community, reflectance quantities recorded by scientists will continue to be study- or site-specific which will in turn limit their broad application and scientific credibility. The issue of standardisation in terminology is of particular importance to spectral measurements collected in EC settings because the primary aim of such measurements is to apply them to the scaling of flux measurements.

### 3.4. Spectral Sensors in European EC Sites: Instrument Characteristics and Set-Up

**Figure 2.**
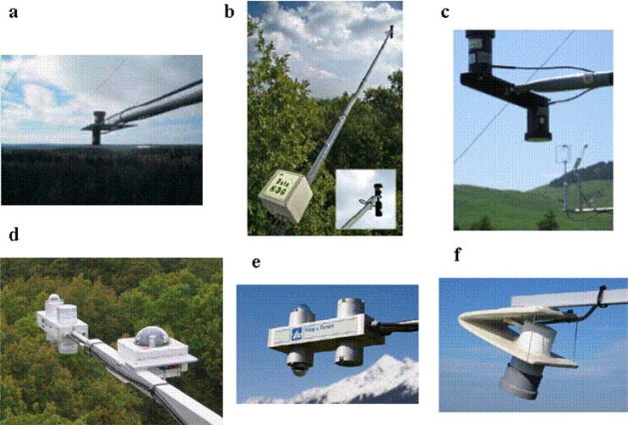
Some examples of the use of 2- and 4-channel sensors at eddy covariance sites in Europe. **(a)** PRI Skye sensor at Hyytiälä forest site (FI-Hyy, Finland), **(b)** PRI Skye sensor at Roccarespampani old forest site (IT-Ro2, Italy), **(c)** NDVI Skye sensor at Monte Bondone grassland site (IT-MBon, Italy), **(d)** Kipp&Zonen sensor at Fontainebleau forest site (FR-Fon, France), **(e)** Kipp&Zonen sensor at Neustift grassland site (AT-Neu, Austria), **(f)** the sensor for monitoring NDVI developed by Jean-Yves Pontailler at ESE Laboratory, CNRS—Univerisité Paris-Sud (France).

**Figure 3.**
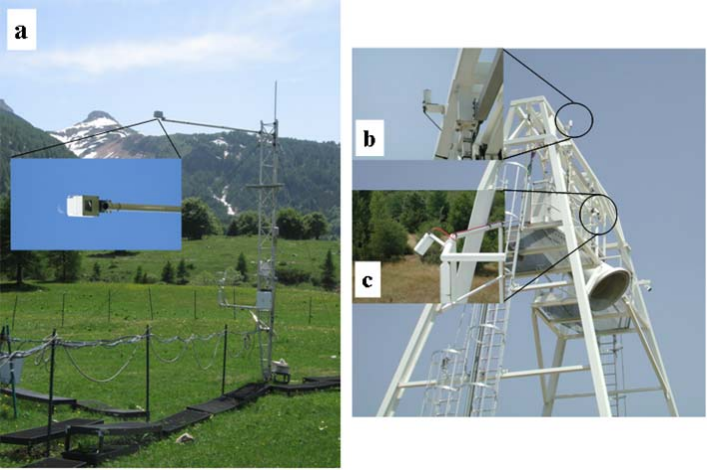
**(a)** Cropscan MSR16R sensor mounted at Monte Bondone grassland site (IT-MBo); **(b-c)** The two CIMEL radiometers mounted at the SMOSREX experimental grassland site (FR-Mau): **(b)** upward sensor, **(c)** downward sensor.

**Figure 4.**
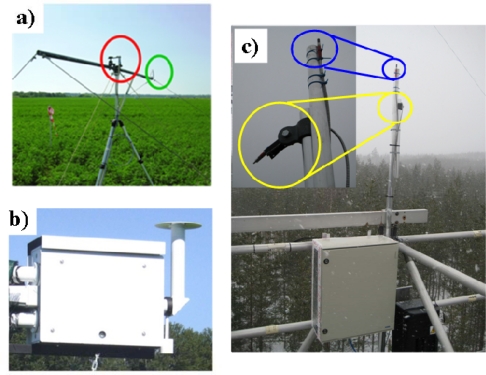
Pictures of the systems installed in the field. **(a)** Optics of the MRI mounted on a tripod (cosine receptor head in red; bare fiber position in green). **(b)** Picture of the HSI box installed in field. **(c)** Two Dual Field of View (DFOV) system installed at Hyytiälä site, Finland (upward pointing cosine receptor in blue; downward fiber in yellow).

The broad range of optical sensors offers a wide assortment of detectors, optics, spectral range and spectral sorting filters, allowing customizable bandsets for a range of environmental applications. This has generated a suite of different methodologies for sampling and interpreting optical data. Continuous measurements are predominantly collected using a nadir view (*i.e.*, 90 degrees to the surface normal) at a single fixed point (Table 1 and Figures 2–4); there are only nine sites that collect off-nadir measurements for estimation of bidirectional reflectance quantities. At most sites there is no consistency in the heights that sensors are installed above the canopies, driven largely by the different tower infrastructures at each site, and by differences in vegetation structure.

Similarly, sensor field-of-view (FOV) used at EC sites varies considerably from site to site, being dependent on the type of sensor and the quantities being measured. For example, some sites use bi-hemispherical instruments with cosine diffusers on both up- and down- looking sensors (e.g., at Monte Bondone (Italy) [8]). Kipp&Zonen and HyperSpectral Irradiometer (HIS) sensors are set up with hemispherical FOVs similarly. In Skye, Multiplexer based Radiometer Irradiometer (MRI), and Cropscan sensors, the FOV is between 25°–28°, while Pointailler’s sensor has an FOV of 60° [19]. The survey showed that the majority of spectral measurements collected at EC sites are logged automatically through time, usually at time intervals of 30 min or less. Optical sensors are initially calibrated by the suppliers and only periodically recalibrated to correct the data drifting and bias from true variations by sending the instruments back to the suppliers.

**Figure 5.**
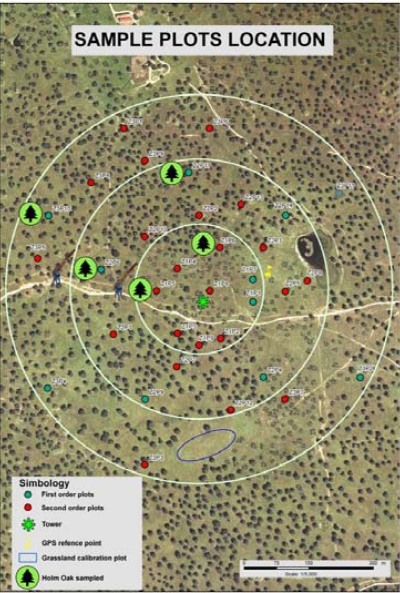
Scheme of Biospec protocol for optical and biophysical sampling.

In some cases continuous automatic measurements are complemented with specific field campaigns. This is the case for the Biospec project (http://www.lineas.cchs.csic.es/biospec) where both optical and biophysical measurements (biomass, water content, nitrogen, chlorophyll) of vegetation cover are collected at a FLUXNET site located at Las Majadas del Tietar (Cáceres, Spain). The area is Mediterranean woody grassland which is grazed by cattle during the vegetation period. The vegetation is composed mainly of short grassland and holm oak trees. In this area twelve 25 × 25 m plots randomly distributed within the EC tower footprint (Figure 5) are sampled every 16 days coinciding with the Landsat 5TM overpass. Destructive vegetation sampling of tree leaves and herbaceous vegetation is carried out for quantifying vegetation biophysical parameters. Additionally, an ASD FieldSpec^®^ FR3 (www.asdi.com) spectrometer is being used to acquire spectral data in the plots. These measurements are taken along two transects with direction NE-SW and NW-SE in each 25 × 25 m plot at the maximum sun elevation (±2 hours from solar noon).

#### 3.4.1. Details on Two and Four Channel Sensors

**Figure 6.**
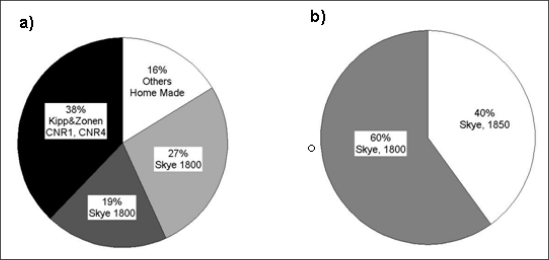
Percentage of 2 and 4-channel sensors used for spectral measurements at the eddy covariance sites. **(a)** 2 and 4-channel sensors for the measurements of NDVI. **(b)** 2 and 4-channels sensors for the measurements of PRI.

Table 1 provides information concerning the most widely used 2- and 4-channel sensors for measuring NDVI and/or PRI at the EC sites. Figure 6 shows the percentage of sites where these sensors are used to measure NDVI or PRI. Two and 4-channel radiometers mounted on EC towers provided by Skye or by Kipp&Zonen are dual-beam. Continuous measurements of NDVI were made at 35 (87.5%) sites; the majority of those sites (38%, 14 sites) used the CNR-1 or CNR-4 Kipp&Zonen to derive a broadband NDVI as proposed by Huemmerich and colleagues [19], 27% (10 sites) the Skye SKR 1850, while 19% (7 sites) used the Skye SKR 1800 and the remaining six sites used specially designed sensors. In some cases (four sites) the CNR-1 Kipp&Zonen and Skye sensors are used together. It is interesting to note that Skye sensors can be configured to use different filters focused on different central wavelengths and bandwidths, while universally maintaining the same optics. In contrast, the characteristics of the Kipp&Zonen sensors are not user selectable, so data are more easily compared between sites. Continuous measurements of PRI using 2- or 4-channel radiometers are collected at 15 sites (37.5%) mainly using Skye instruments (SKR-1800, SKR-1850): 60% (9 sites) of those sites use the 2-channels SKR-1800 sensor and the remaining sites used the 4-channels SKR-1850 sensor (Figure 6). Both Skye and Kipp&Zonen sensors are directly calibrated at the factories. Skye calibrates the sensors against a National Physical Laboratory UK reference standard lamp, while Kipp&Zonen sensors uses the World Radiometric Reference standards. Continuous measurements of both NDVI and PRI are predominantly collected mounting the sensor at the top of the flux tower looking at a single fixed point. Only in the Gungahlin site in Australia, data are collected at various points over a given sampling area using two nodes, each with paired sensors. Data are automatically logged through time every 30, 10 or 1 min. Figure 2 shows some examples of set-ups employed at the eddy covariance sites in Europe utilizing Skye sensors (a–c) and Kipp&Zonen sensors (d–e).

At Lanjaron, El Saler-Sueca, Vall d’Alinya and Corte de Pallas sites in Spain and at Hesse and Fontainebleau sites in France, measurements of NDVI are collected by custom NDVI sensor built by Pontailler and colleagues [19] at ESE Laboratory of CNRS in collaboration with the University of Paris-Sud. This laboratory-made sensor is a dual beam sensor working in the red (640–665 nm) and near-infrared (750–950 nm) bands. It is equipped with two photodiodes having a large photosensitive surface to ensure a high sensitivity that measure radiances in the red region around 655 nm and in the NIR around 825 nm. For the red channel a gallium arsenide phosphide photodiode is used, while the NIR channel uses a silicon photodiode and a longpass glass filter (for more details see [19]). Data are automatically logged each 30 min. The sensor is mounted in a fixed position at the top of eddy covariance flux tower; therefore, the optical sampling is made at single fixed point. The extension of the footprint of the optical measurements varies with the sensor height over the canopy that is site specific. Figure 2(f)) shows the sensor mounted at the Fontainebleau forest site.

#### 3.4.2. Multispectral Sensors

A dual beam Cropscan MSR16R sensor (Cropscan, USA) is used at the Monte Bondone EC site to measure Hemispherical Conical Reflectance Factors. Upward and downward facing sensors measure both incoming and reflected radiation, nearly simultaneously, which allows for useful reflectance readings in lightly cloudy conditions down to about 300 W/m^2^ incident global radiation. The sensor is mounted in fixed position at 6 m above the canopy and the FOV of downward looking optic is 28°. Data are automatically logged each 10 min from 9.00 AM to 13.00 PM UTC. Cropscan is calibrated by researchers of Monte Bondone each year following the calibration methodology available in the manual device (http://www.cropscan.com/2ptupdn.html). Figure 3(a) shows the sensor mounted at the Monte Bondone grassland site.

At the SMOSREX experimental grassland site (FR-Mau) the Hemispherical-Conical Reflectance Factor is determined using two CIMEL single beam radiometers (Cimel Electronique, FR, Europe) [31]. Reflectance is detected at five wavelengths in the visible and infrared regions: 450 nm (±20 nm), 549 (±42.5 nm), 648 nm (±26.5 nm), 837.5 nm (±45.5 nm), 1,640.2 nm (±82.3 nm). Both sensors are positioned at 15 m above the canopy viewing a fixed area. The downward sensor is looking southward and the upward sensor has an angle of 0° from horizon, while the downward sensor is placed with angle of 40°. A cos-conical calibration method, which calibrates according to an upward-pointing irradiance sensor, was applied in 2003 to calibrate the radiometer. Reflectance readings are automatically collected at a single fixed point and logged every 60 min. Figure 3(b,c) show the set-up of the two CIMEL sensors mounted at SMOREX site.

#### 3.4.3. Hyperspectral Sensors

Few sites are equipped with automatic spectral systems for the collection of unattended, continuous, long time series of hyperspectral measurements at the moment. The hyperspectral systems currently installed at European EC sites are: the Multiplexer based Radiometer Irradiometer (MRI) [10] and the HyperSpectral Irradiometer (HIS) [32] developed at the University of Milano-Bicocca (Italy). Both of these systems utilize Ocean Optics spectroradiometers. In addition other two Ocean Optics spectrometer systems are used in Europe. One system developed by Nichol and colleagues in the School of GeoSciences at the University of Edinburgh is deployed at Hyytiälä (Finland), and the other (TriFLEX) developed by the Centre National de la Recherche Scientifique, Ecole Polytechnique of Palaiseau Cedex is deployed at Avignon (France) site [33].

The MRI is based on a commercially available optical multiplexer (MPM-2000, OceanOptics, USA) which is able to switch between a channel measuring incident irradiance (cosine response foreoptic), a down-looking bare fiber (field of view of 25°) for the measurement of upwelling radiance and a “blind” channel for the spectrometer dark current measurement. MRI thus allows the measurement of the HCRF. Optical components (multiplexer, spectrometers), personal computer and power supply are housed in a thermally controlled protective box to ensure that the radiometric response is stablised through time, and to reduce measurement uncertainty associated with changes in ambient conditions [36]. The HSI system uses a rotating cosine-response optic to measure the irradiance incident on and upwelling from the investigated surface, allowing the computation of the bi-hemispherical reflectance (BHR). The spectrometer dark current is measured at each acquisition session using a mechanical shutter. Among other devices available on the market, OceanOptics (Dunedin, FL, USA) spectrometers were chosen because they are small, highly configurable in terms of spectral range and resolution and relatively inexpensive. Spectrometers embedded in both HSI and MRI are spectrally calibrated with a known standard (CAL-2000 mercury argon lamp, OceanOptics) and radiometrically cross-calibrated to a FieldSpec FS FR spectrometer (ASD, USA) which in turn is calibrated by the manufacturer with a yearly frequency. Furthermore, the stability of the spectral calibration is regularly assessed using field measured irradiance data and the SpecCal algorithm [12]. Both systems are operated automatically by a personal computer through the 3S software [37]. An HSI system is currently installed on a flux tower in Torgon (IT-Tor, Italy) and a MRI on a flux tower near Pisa (IT-Pisa, Italy). Figure 4 gives an example of the use of MRI (Figure 4(a)) and HSI (Figure 4(b)). The method used by this group is among the most rigorous of all those surveyed, and is a worthy benchmark for reproducible measurements for others to follow.

Nichol and colleagues at the University of Edinburgh have similarly designed, built and are running two Dual Field of View (DFOV) systems. The rationale for the deployment of these new optical systems is to utilize remotely developed methods to detect changes in photosynthesis at the canopy scale through the integration of tower, aircraft and satellite-based remotely sensed data. In doing so the project is actively describing the temporal and spatial dynamics of photosynthetic light use efficiency (ε) and chlorophyll fluorescence (CF) in contrasting vegetation types in a north-to-south climate gradient. Upward and downward-pointing rugged spectrometers (Ocean Optics USB2000+, FMHM 1.0 nm, spectral range 400–1,000 nm,) housed in thermally controlled boxes, (maintaining internal temperature at 35 °C + 0.5 °C) are currently installed on two flux towers (Hyytiälä, Finland and Griffin, UK) and are acquiring spectral data continuously at between 5–15 min intervals (Figure 4(c)). An upward pointing cosine fiber optic measures irradiance and a downward pointing 600 micron fiber measures upwelling radiance. The spectral data are acquired simultaneously and are intercalibrated. In house (stable) software has been developed (in Java) and controls the operation of each pair of spectrometers.

TriFLEX uses two identical spectrometers (HR2000+, Ocean Optics) to simultaneously measure irradiance and vegetation radiance spectra [33]. These spectrometers cover the spectral range of 630–815 nm with a resolution of 0.5 nm [full-width at half-maximum (FWHM)] and an encoding resolution of 0.09 nm/pixel. The main advantage of simultaneous measurement of the reference and the sample is an improved time resolution by a factor of two to three, which allows for fast fluorescence changes induced by clouds and sun spells to be monitored. A third spectrometer (HR2000+, Ocean Optics) measures vegetation radiance on the spectral range 300–900 nm (50 μm entrance slit, FWHM-2 nm). Every 20 min, the reference board is switched from the default position to the calibration position by the means of a rotary solenoid (GDAX035X20E06, Magnet-Schultz). In the calibration position, the reference intercepts the FOV of all spectrometers. At the end of the day, a linear relationship is deduced from the measurements of vegetation and reference.

### 3.5. Applications

The development of multispectral and hyperspectral sensors for continuous measurements of canopy optical properties opened new insight on vegetation indices applications in ecological modeling, biophysical parameters assessing (LAI, biomass, nitrogen and water content) and carbon fluxes estimating. Several authors proposed the use of multispectral optical sensors to derive some broadband and fine multispectral vegetation indices, as NDVI and SR [18,38–40]. Also Wohlfahrt and colleagues [9] that filled the questionnaire used this approach based on broad-band NDVI sensors to estimate carbon dioxide fluxes of temperate mountain grasslands in Austria. Tower-derived NDVI values are closed to *in situ* NDVI [23,38] but the variability of tower-NDVI is not as high as in the radiometer *in situ* NDVI. Wilson and Meyers [39] and Tittedrant *et al.* [23] reported that tower-NDVI shows close values to MODIS satellite data for grassland and crops but large scatter for forests. The advantage of the broadband NDVI is their temporal resolution that can be hourly or higher and the absence of the atmospheric disturbances. They are also low cost sensor and easy to use responding thus to limited funding available in most of cases for EC groups. Abergel and collaborators used continuous reflectance measurements of fine multispectral resolution of CIMEL sensors for predicting LAI and validate SURFLEX model outputs [31].

Regarding the use of hyperspectral sensors at EC sites, there are still few scientific teams that are working on this issue. These groups focused their attention mainly on studying florescence, estimating PRI and modeling ecosystem gross production. Beyond our specific survey results, it is important to highlight the relevant works of Hilker *et al.* [16,17] and Gamon *et al.* [13] who have developed novel methods for year-round unattended hyperspectral measurements at flux sites with excellent results. The power of these approaches is the application of methods for estimating spatially coherent vegetation properties which hold meaning for the EC footprint, rather than just for a single fixed point below the tower. In Europe, Rossini and colleagues [10] are leading the way in developing hyperspectral systems with good reproducibility. The reason for the small number of people using hyperspectral systems in unattended settings is that hyperspectral spectroradiometers are not necessarily designed with this in mind, and therefore adapting them to be weather proof, motorized and with data storage connections is not trivial. Technological advances in recent times by those mentioned above will pave the way for a larger scientific deployment of these instruments across other sites, because the results obtained are already showing good relationships with EC fluxes [9,10,20,33,40].

## 4. Current Controversial Issues

From the responses to the online survey it is possible to answer to which optical sensors are used by the EC community and how these data are collected but there are still some open questions linked mainly to spatial and temporal resolution, scale-appropriateness of measurements, long-term data and sensor calibration, and data repeatability and reproducibility that require further discussion.

### 4.1. Radiometric and Spectral Resolution

In general, dual beam configuration, where reflectance measurements are made nearly instantaneously, is known to produce more precise data than single beam systems, because of the reduced time delay between reference measurements of the incident irradiance, and measurements of upwelling radiance from the target. The caveat is that for this to work, both dual-beam instruments must be spectrally and radiometrically matched and inter-calibrated [41] which carries an additional processing and cost burden.

Hyperspectral and fine spectral resolution multispectral sensors allow for the quantitative analysis of spectral features of the canopy biophysical variables (e.g., biomass, water, nitrogen and chlorophyll content, and photosynthesis pigment activity) to be carried out [42–44]. The main limitation is that these sensors are usually configured for just one specific index and therefore flexibility to calculate other indices is limited.

The great advantage of using hyperspectral sensors rather than multispectral sensors is thus the possibility to use the full spectral information and to compute any desired vegetation index rather than a pre-defined one dictated by the sensor spectral set-up. Moreover the selection of new narrow wavebands in hyperspectral data has demonstrated an increase in the sensitivity of the vegetation indices to different vegetation biophysical variables [45]. Hyperspectral instruments are installed in a limited EC towers for continuous/unattended measurements [10,17,25,32] due to the high start-up costs and the additional complexity of making them weatherproof and automated.

### 4.2. Spatial Resolution (Footprint)

The spatial dimension of spectral measurements at EC sites (e.g., the connection between the sensor support and the EC footprint) continues to be an area of great debate. This issue is related to the spatial representativeness of spectral data acquired in complex ecosystems where plant architecture, density and homogeneity play an important role in both the spectral response of the canopy, and in the gaseous fluxes measured at EC towers. With regards to this, the main scientific challenge is to identify the most suitable spatial support for optical sampling in flux settings and to define how this can be considered to be statistically representative of vegetation types and their physiological/ phenological status. This issue is strictly linked to the extension of both fluxes and radiation measurements and also to the EC footprint [46,47]. It is well known that flux footprints vary in relation to atmospheric stability, wind speed, measurement height, canopy structure and the vertical distribution of sources and sinks. For example, the dimension of the source area of turbulent fluxes can vary between 10 to 100 times of the measurements height during the same day as a result of changes in micro-meteorological conditions [46]. On the contrary, the relationship between the footprint of radiation sensors and the flux footprint has not fully been considered and is necessary to take it into account when using datasets from both EC sensors and spectral measurement systems in tandem. For this reason, the footprint of the radiation sensors must be fully characterized prior to use through reference to the physical capabilities of the instrument (e.g., FOV and optics), in combination with an understanding of how and where the sensor is mounted on the flux tower (height of measurements, orientation). Orienting the sensor obliquely will increase the footprint area of the radiation measurements, and will also increase the proportion of projected canopy to ground. All directional measurements are affected by reflectance anisotropy, which needs to be considered when orienting the sensor. From the responses to the questionnaire, it is clear that optical samplings at the flux tower are usually collected at a single fixed direction. In the literature there are some studies where optical measurements are made considering the eddy fluxes footprint. For example in Hilker *et al.* [16,17] optical spectral measurements were collected over a circular area centered at the flux tower using a rotating system while Gamon and colleagues [13] used a tram system to make spatially representative measurements within the EC tower footprint. These are really unique systems that fully consider the relationship between the measurement support of the spectral instrument and the EC footprint.

### 4.3. Distribution of Light throughout the Canopy (BRDF Problem)

Another fundamental aspect to be considered in optical sampling is the distribution of light throughout the canopy (*i.e.*, the classic surface BRDF problem). Reflection, absorption and transmission of sunlight depend on canopy structure, foliar distribution and solar zenith angle. Only the system proposed by Hilker and colleagues, AMSPEC-II system, is integrated with a LiDAR device that permits also the measure of the light transmitted into the canopy [17]. AMSPEC is designed to automatically acquire spectra with different viewing angles allowing the analysis and interpretation of the optical signals in terms of BRDF. However complementary measurements made by LiDAR [17] should help to better understand the role of plant architecture, density and homogeneity in the vegetation spectral responses. There is still considerable work to do on this subject and our understanding of how best to tackle this problem at EC sites requires further work.

### 4.4. Calibration

The calibration of automated optical sensors used to monitor flux sites on a continuous basis is a new challenge within the remote sensing community. Our survey showed that some of the spectral instruments used at EC sites were rarely calibrated against standard laboratory sources. This is probably because in “reflectance” scenarios, the absolute radiometric calibration of the instrument is not critical due to the ratioed nature of the reflectance factor calculation. While we would agree that absolute radiometric calibration of sensors (e.g., to radiance or irradiance) is less important if reflectance quantities are being measured, three key elements of the measurement are critical: 1. the absolute reflectance of the calibration standard (e.g., Spectralon panel) in bi-conical measurement situations; 2. the cosine response of any irradiance sensor used in cos-conical methods; and 3. regular wavelength calibrations of all sensors used. The first two are central to the magnitude of the determined reflectance quantity being correctly defined, and the latter is needed to ensure that any spectral indices determined are using the correct wavelength regions. All users of such equipment could perform simple in-field or dark-room checks to enable early detection of sensor or panel calibration drift but our survey did not give clear results to show whether this was being frequently undertaken by users. Failure to ensure regular calibration of these instruments will result in non-reproducible results which will be of limited scientific value.

### 4.5. Temporal Resolution

Temporal and spatial sampling issues remain the key aspects in the framework of optical and flux data matching in traditional vegetation studies. Standard field spectroscopy generally consists of periodically taking series of point measurements of vegetation (leaf and/or canopy) at times of the day when the solar angle does not cause excessive shadowing (*i.e.*, ±2 hours of solar noon) throughout the vegetation period. In this framework, continuous ground measurements are crucial to overcoming the mismatch problems with the temporal scale of the satellite data collection [18].

### 4.6. Repeatability, Reproducibility and Cross-Comparison of Measurements

A further challenge for researchers involved in optical sampling is how to make repeatable and reproducible spectral measurements. In order to compare field spectral measurements between different instruments, or across different field sites, it is first necessary to understand the physical capabilities of the sensors. For example, repeatability measures such as the noise equivalent delta radiance (NEΔL; measured in laboratory conditions, usually against an integrating sphere source) can inform about the baseline sensitivity of the radiometer’s detector(s) across the spectral range. Such data convey information on the radiant sensitivity of the detector, and thus on the most reliable region of the spectrum to sample [48,49]. Regular wavelength checks on instruments as well as wavelength calibration are also important, particularly for vegetation applications. This is because algorithms such as PRI require the delineation of narrow spectral features, which relies on precise definition of the wavelength position. Offsets or shifts in the instrument’s wavelength sensitivity could yield misleading results in such applications. It is important to bear in mind that the above two measures yield only a laboratory-derived quantification of instrument precision and in isolation they are unlikely to describe all the uncertainty associated with field spectral measurements. It should also be observed that field-sensors are commonly affected by dark-current drift which is a temperature-dependent shift in values that may vary across the season, particularly at sites with large temperature variations [32]. This shift can be corrected using night-time values in continuous monitoring systems [29] or using automatic dark current shutters in sensors such as ASD FieldSpec Pro. For cross-comparison across sites, or between different instruments, a more detailed assessment and documentation of data quality is required. Here we explicitly refer to the “reproducibility” of the measurements, which is dependent upon the spectroradiometer’s inherent NEΔL and wavelength sensitivity, coupled with the methodology used to acquire the data under field conditions. This will, of course, vary from instrument to instrument, and according to the specific conditions prevailing at the time of the measurement (e.g., the solar zenith angle, sky conditions, time of day etc, and including the instrument set-up and the target being measured). There is now a drive for all users of spectroradiometric equipment to both measure and document reproducibility estimates for published measurements [48] so that such data have long-term value beyond the scope of the individual studies.

## 5. Conclusion and Issues for the Future

This review has shown that in the EC community there is no consensus about a single methodology to collect optical measurements at EC sites. At the moment, groups of researchers undertaking these measurements are typically operating individually, following their own methodologies for such measurements. This has resulted in a range of different instruments and approaches for spectral measurements at EC sites. The choice of the sensor and their use is principally linked to available budget that in most cases is limited because of principal investigators must self-fund the acquisition, set up and processing of spectral data satisfying individual funding applications. This creates challenges for comparisons between sites, and limits the possibility of developing a common protocol for use at all EC sites. However, a helpful initiative on the integration and standardization of optical sampling into European EC network it represented by IMECC-“*Infrastructure for Measurements of the European Carbon Cycle*” (more details at http://imecc.ipsl.jussieu.fr). The innovative aspect of IMECC with respect to previous projects was to propose the use of same sensor and following the same methodology to measure spectral response in the photochemical reflectance index (PRI) [50] bands. So far IMECC has supported the development of ICOS-“*Integrated Carbon Observation System*” initiative (more details at http://www.icos-infrastructure.eu/) that aims to establish an integrated long-term research infrastructure for understanding the biogeochemical cycles of greenhouse gases in Europe. Currently ICOS is in the preparatory phase (2009–2012) during which the set-ups of the sensors and protocols of measurements at the eddy covariance sites are defined. Analysis of optical samplings needs and limits discussed in this review can act as a first line guide on optical sapling at EC helping researchers to choose the most suitable sensor and set-up to use in their sites (e.g., in the ICOS framework). From this review two possible standards (*basic standard and advanced standard*) could be considered in relation to IMECC and ICOS requirements, with regards to recommending the best approach for optical measurements at EC sites.

### 5.1. The Basic Standard

In the *basic standard* set-up the main consideration is the cost-effectiveness of the instrument in delivering routine high-quality radiometric data for year-round unattended measurements. This standard could be adopted at a large number of sites in order to provide high quality reflectance factor data on reflectance quantities needed for cross-comparison. Our survey showed a wide use of (comparatively) low-cost (e.g., Skye) and radiometrically sensitive multispectral sensors for continuous measurements at EC sites. These sensors have proven capabilities in measuring NDVI and PRI. Scientific evidence points to these being fit for purpose and able to withstand the rigours of continuous exposure to weather. The main issue of the review is that users do not consistently check their calibration, and this would be required for maintaining high data quality across sites. Additionally ICOS and IMECC would require that these sensors be settled-up according to uniform measurement geometry and that measurement acquisition be standardized with respect to timing, calibration and data processing.

There are several options then to consider: Skye fine resolution multispectral radiometers or broadband radiometers (Hukseflux, Delta-HOM, Kipp&Zonen). Each has its relative merits, Skye sensors are more flexible in their ability to be configured towards collection of data for specific spectral indices (e.g., NDVI, PRI). Moreover, using a 4-channel Skye instrument it is possible to concurrently measure two vegetation indices. The upwards-pointing cosine diffuser however, would need to be regularly tested to ensure a good cosine response. The other radiometers (*i.e.*, Hukseflux, Delta-HOM, Kipp&Zonen) are easy to deploy but require an additional sensor to provide reflected global radiation so that NDVI can be calculated.

### 5.2. The Advanced Standard

The *advanced standard* could be proposed for a limited number of sites where high standard of all flux and meteorological variables are required. In this case we recommend to add to standard instruments package an instruments for hyperspectral measurements that could be the UniSpec-DC (PP Systems, USA) or USB2000 (Ocean Optics) hyperspectral radiometers. However with a large budget supply it should be reasonable to use AMSPEC II systems [17] because it is the most proven existing technology for optical sampling at the EC sites. AMSPEC II can collect optical data with a very high spatial, temporal, and spectral resolution under different view and sun angles and allows a complete analysis of the relationships between spectral reflectance and carbon flux dynamics.

### 5.3. General Guidelines for Routine Optical Sampling at EC Sites

Optical sampling at the EC sites should be made following the recommendations reported here. For routine optical measurements it is enough to use multispectral sensors indicated in the *basic standard* procedure. We recommend using dual beam fine resolution (e.g., Skye) or multispectral sensors (*i.e.*, Hukseflux, Delta-HOM, Kipp&Zonen). The field of view of downward sensors could vary between 20 and 25 degrees for fine resolution multispectral sensors or nearly to 180 degrees for multispectral sensors. In both cases the optic of the up-looking sensor could be a cosine receptor. To increase footprint of optical measurements and proportion of projected canopy to ground nadir position, we recommend orienting the sensor obliquely and defining sensor’s height over the canopy considering FOV of the sensors (footprint) together with structure and spatial distribution of the vegetation. Using fine resolution multispectral sensors, spectral bandwidths for NDVI should be lower than 50 nm [22] centered roughly at 680 nm and 760 nm while for PRI bandwidths for NDVI should be lower than 10 nm centered at 529 nm and 568 nm. We recommend cleaning and checking always the right position of the sensors. Calibration of the sensors should be necessary at least ones per year at the beginning of the growing season by sending back sensor to the suppliers. The optical data should be collected at least each 30 min agreeing with time resolution of fluxes and meteorological variables even if a higher time resolution is more suitable in understanding photosynthetic processes and light response [50]. All optical data should be stored with other energy fluxes variables as incoming and outgoing radiation and diffuse radiation that can help to understand environmental condition during spectral acquisition. Finally all changing in set-up or in data acquisition should be reported in order to check the quality of data.

## Figures and Tables

**Table 1.** List and principal characteristics of the automatic optical systems currently installed at European eddy flux towers. The site name, site FLUXNET ID, optical system (Manufacturer/Model), spectral range/channels (nm) and their resolution (FWHM, nm), the method used for acquisition (Single/Dual beam), the geometry of acquisition (Nadir/Off-Nadir/MultiViewAngle), the spatial sampling scheme (Single point/Transect/Multi point), the reflectance quantity (BHR, Bi-Hemispherical Reflectance factor/HCRF, Hemispherical-Conical Reflectance Factor), the downward Field of View (FOV), sensor Height Above the Canopy (m) and references, when available, are reported. K&Z stands for Kipp&Zonen. HM stands for home made. “na” stands for answer not available.

**Site Name**	**Site ID**	**Optical system/ Manufacture/ Model**	**Spectral range/ channel center (Spectral resolution/FWHM) [nm]**	**Time resolution (averages) [min]**	**Method Single/Dual beam**	**Nadir/Off-Nadir/ MultiViewAngle [°]**	**Single Point /Transect/ Multi point**	**Reflectance Quantity**	**Downward FOV [°]**	**Height Above Canopy [m]**	**Ref.**
***2- and 4-channel sensors***											
Neustift, AT	AT-Neu	K&Z CNR1	300–2,800	30	D	N	SP	BHR	cosine	0.5–1.5 (depending on canopy height)	[9]
Hesse, FR	FR-Hes	HM SKYE 1800	645 (628–680), 780 (731–998) 530 (7), 569 (8)	30 30	D D	N N	SP SP	HCRF HCRF	60 25	2.5 2.5	[27]
Laqueuille, FR	FR-Lq1	SKYE 1800 SKYE 1800	red (na), NIR (na) 529, 568 (8)	30 30	D D	N N	SP SP	HCRF HCRF	25 25	2 2.5	
Loobos, NL	NL-Loo	SKYE 1800	530, 569 (7)	30	D	N	SP	HCRF	25	6.0	
Las Majadas del Tietar, ES	ES-LMa	SKYE 1850	530 (7), 569 (7), 679 (12), 798 (12)	10	D	N	SP	HCRF	25	12 (grass) 5 (oak)	
El Saler-Sueca ES	ES-ES2	HM	660 (15), 820 (775–900)	10	D	ON (20)	SP	HCRF	60	1.5–2.5 (depending on canopy height)	
Vall d’Alinya, ES	ES-VDA	HM	660 (15), 820 (775–900)	10	D	ON (20)	SP	HCRF	60	na	
Cortes de Pallas, ES	ES-CPa	HM	660 (15), 820 (775–900)	10	D	ON (20)	SP	HCRF	60	na	
Puechabon, FR	FR-Pue	K&Z CNR1 SKYE 1800	300-2800 531, 570 (na)	30 30	D S	N ON	SP SP	BHR HCRF	cosine 25	6.0 6.0	
Lanjaron (PN. Sierra Nevada), ES	Lanjaron *	HM	655 (640–660), 825 (780–950)	30	D	N	SP	HCRF	60	na	
Collelongo, IT	IT-Col	K&Z CNR1	300–2,800	30	D	N	SP	BHR	cosine	4.5	
Bonis, IT	IT-Bon	K&Z CNR1	300–2,800	30	D	N	SP	BHR	cosine	3.5	
Boschi di Carrega, IT	ICP-Forests IT0005 (EMI1-Carrega) *	K&Z CNR1	300–2,800	30	D	N	SP	BHR	cosine	4.5	
Monte Bondone, IT	IT-MBo	SKYE 1850	550 (10), 680 (10), 749 (20), 849 (20)	1	D	N	SP	HCRF	25	1.0–1.5 (depending on canopy height)	[8]
Lavarone, IT	IT-Lav	SKYE 1800	665 (70), 843 (125)	1	D	N	SP	HCRF	25	10.0	
Valle dell'Adige, IT	IT-VdA	SKYE 1850	549 (9.4), 678 (11.7), 750 (20), 849 (18.6)	1	D	N	SP	HCRF	25	5.0	
Roccarespampani young forest, IT	IT-Ro1	K&Z CNR1	300–2,800	30	D	N	SP	BHR	cosine	5.0	
Roccarespampani old forest, IT	IT-Ro2	K&Z CNR1 SKYE 1800	300–2,800 529, 568 (8)	30 1	D D	N N	SP SP	BHR HCRF	cosine 25	3.0 5.0	
Amplero, IT	IT-Amp	K&Z CNR1	300–2,800	30	D	N	SP	BHR	cosine	3.5–4.5 (depending on canopy height)	
Renon, IT	IT-Ren	K&Z CNR1 SKYE 1800	300–2,800 red (na), NIR (na)	30 30	D D	N N	SP SP	BHR HCRF	cosine 25	10.0 10.0	
Fontainebleau, FR	FR-Fon	HM SKYE 1800	660 (15), 820 (775–900) 531, 570 (10)	30 30	D S	ON (20) ON (20)	SP SP	HCRF HCRF	60 25	na na	
Wicken Fen, UK	FENFLUX *	K&Z CNR1	300–2,800	30	D	N	SP	BHR	cosine	0.5–2 m (depending on canopy height)	
Rzecin wetland, PL	PL-WET	K&Z CNR1 SKYE 1850	300–2,800 530, 570, 670, 850 (10)	30 30	D D	N N	SP SP	BHR HCRF	cosine 25	2.5 .5	
Tuczno forest, PL	PL-FRT	SKYE 1850	530, 570, 670, 850 (10)	30	D	N	SP	HCRF	25	8.0	
Brody arable, PL	PL-ARB	K&Z CNR4 SKYE 1850	300–2,800 530, 550, 570, 670, 750, 850, 900, 970 (10)	30 30	D D	N N	SP SP	BHR HCRF	cosine 25	3.0 3.0	
Yenicaga Peatland, TR	CAYDAG 109Y186 *	K&Z CNR4 SKYE 1800 SKYE 1850	300–2,800 530, 570 (10) 455–520 (65), 630–700 (70), 760–900 (140)	60 30 30	D D D	N N N	SP SP SP	BHR HCRF HCRF	cosine 25 25	3.0 3.0 3.0	[28]
Herdade da Machoqueira, PT	PT-Cor	SKYE 1800	531, 570 (5)	30	D	ON	SP	HCRF	25	∼7.0	
Hyytiälä, SMEAR II NDVI, FI	FI-Hyy	SKYE 1800 SKYE 1800	529, 568 (8) 652 (54), 861 (53)	1 30	D D	ON (55) ON (55)	SP SP	HCRF HCRF	25 60	15.0 15.0	[29]
Abisko Delta forest, SE	Abd1 *	SKYE 1850	649, 867, 528, 568 (11)	10	D	ON (55)	SP	HCRF	60	9.0	[29]
Abisko Stordalen mire, SE	Abs1 *	SKYE 1850	649, 867, 528, 568 (11)	10	D	ON (55)	SP	HCRF	60	8.0	[29]
Fajemyr mire, SE	SE-Faj	SKYE 1800	652 (54), 861 (53)	10	D	ON (55)	SP	HCRF	60	10.0	[29]
Norunda forest, SE	SE-Nor	SKYE 1800	652 (54), 861 (53)	30	D	ON (55)	SP	HCRF	25	30.0	[29]
Zackenberg arctic fen, DK	DK-Zaf *	SKYE 1800	655 (48), 855 (55)	30	D	N	SP	BHR	cosine	2.0	
Skukuza, ZA	ZA-Kru	K&Z CNR1	300–2,800	30	D	N	SP	BHR	cosine	na	
Gungahlin Australia	- *	SKYE 1850	545 (10), 650 (11), 833 (12), 1,047 (14)	1	D	N	MP	HCRF	25	1.5	[30]
***Multispectral sensors***											
Monte Bondone, IT	IT-MBo	Cropscan MSR16R	467 (10), 530 (8.5), 546 (10), 570 (10), 610 (10.3), 640 (11.3), 680 (10), 720 (12.6), 730 (12.9), 750 (13.4), 780 (10), 860 (10), 900 (12.7), 1,240 (11.6), 1,660 (15.6)	10	D	N	SP	HCRF	28	∼5.7	
SMOSREX, FR	FR-Mau	CIMEL	450.0 (430.0–470.0), 549.0 (506.5–591.5), 648.0 (621.5–674.5), 837.5 (792.0–883.0), 1,640.0 (1,557.7–1,722.5)	60	D	ON (40)	SP	HCRF	na	15	[31]
***Hyperspectral sensors***											
Torgnon, IT	IT-Tor	HSI, OO, HR4000 (Ocean optics) HSI, OO, HR4000 (Ocean optics)	400–1,000 (1) 700–800 (0.1)	5 5	S S	N N	SP SP	BHR BHR	hemispherical hemispherical	3.5 3.5	[32]
San Piero a Grado, Pisa, IT	IT-Pisa*	MRI, OO, HR4000 (Ocean optics) MRI, OO, HR4000 (Ocean optics)	400–1,000 (1) 700–800 (0.1)	3 3	S S	N N	SP SP	HCRF HCRF	25 25	1.2–2 1.2–2	[10,32]
Hyytiälä, FI	FI-Hyy	Ocean optics USB2000+	Ocean optics USB2000+	A (defined by user, min: 1 min max: 1 h)	D	ON (∼35)	SP	HCRF	22	na	
Griffin Forest - Scotland, UK	UK-Gri	Ocean optics USB2000+	Ocean optics USB2000+	A (defined by user, min: 1 min max: 1 h)	D	ON (∼35)	SP	HCRF	22	na	
Avignon, FR	FR-Avi	TriFlex,	Ocean Optics, HR2000+ (630–815–0.5 nm) Ocean Optics, HR2000+ (300–900–2nm)	(<1)	D	N	SP		5	20	[33]

* Not a FLUXNET site.
